# Fulminant Tuberculous Pericarditis Presenting as Cardiac Tamponade in an Infliximab-Treated Patient

**DOI:** 10.7759/cureus.94848

**Published:** 2025-10-18

**Authors:** Shehab Hasan, Omar Elboraey, Sian Josse, Abououf Marwan, Irfan M Ahmed

**Affiliations:** 1 Emergency Medicine, Lancashire Teaching Hospitals NHS Foundation Trust, Preston, GBR; 2 Cardiology, Macclesfield District General Hospital, Macclesfield, GBR; 3 Microbiology, Lancashire Teaching Hospitals NHS Foundation Trust, Preston, GBR; 4 Respiratory Medicine, Royal Preston Hospital, Preston, GBR; 5 Cardiology, Royal Preston Hospital, Preston, GBR

**Keywords:** cardiac tamponade, case report, immunosuppression, infliximab, tuberculous pericarditis

## Abstract

Tuberculous pericarditis (TBP) is a rare but potentially life-threatening manifestation of extrapulmonary tuberculosis (TB), representing a clinically important subset of pericarditis cases. Diagnostic uncertainty arises from variable symptoms and the low sensitivity of available tests. Although culture is the gold standard, it is reliable but too slow to enable a timely diagnosis. Acute and fulminant presentations have been reported in immunosuppressed patients. A 61-year-old woman, recently receiving infliximab and corticosteroids for Crohn’s disease, presented to the emergency department with signs of sepsis and obstructive shock. Echocardiography revealed a large pericardial effusion, prompting urgent pericardiocentesis. Analysis of pericardial fluid was negative for acid-fast bacilli. She remained unwell, with persistently elevated inflammatory markers, bilateral pleural effusions, pulmonary nodules, intra- and extra-thoracic lymphadenopathy, and subsequently developed heart failure. After nearly two weeks, endobronchial ultrasound-guided lymph node aspiration with Cepheid GeneXpert TB PCR confirmed rifampicin-sensitive *Mycobacterium tuberculosis*. This case is unusual because it combines three rare features: fulminant TBP presenting as acute tamponade, reactivation of TB in the setting of anti-tumor necrosis factor therapy, and transient left ventricular systolic dysfunction following pericardial drainage. It highlights the importance of considering TB pericarditis in immunosuppressed patients, recognizing the limitations of conventional fluid-based diagnostics, and pursuing timely tissue sampling to confirm the diagnosis and guide appropriate therapy.

## Introduction

Tuberculosis (TB) remains a major global health problem, with extrapulmonary disease occurring in 15-20% of cases [1]. Approximately 60% of patients with TB develop concurrent cardiovascular involvement, most commonly pericarditis, myocarditis, or coronary artery disease [2]. Pathogenesis typically begins with *Mycobacterium tuberculosis *(MTB) reaching the pericardium via retrograde lymphatic spread from infected mediastinal nodes, hematogenous dissemination, or direct extension from adjacent pulmonary or pleural lesions [2].

Tuberculous pericarditis (TBP) reportedly accounts for less than 1% of extrapulmonary TB cases [3] and approximately 4% of all acute pericarditis cases in high-income countries [4]. Despite its relative rarity in these settings, TBP is a serious condition, with mortality rates ranging from 17% to 40% [4]. A primary contributor to this high mortality is progression to severe complications, such as cardiac tamponade, which occurs in 7% of acute pericarditis cases and is only seldom reported in TBP cases in high-income countries [5].

Anti-tumor necrosis factor-alpha (TNF-α) agents, particularly infliximab, block the activity of tumor necrosis factor α, a cytokine essential for the formation and maintenance of granulomas that contain latent *M. tuberculosis*. When TNF-α is neutralized, macrophage activation is impaired, and the structural integrity of granulomas breaks down, allowing dormant bacilli to proliferate and cause active disease [6,7].

Diagnosis of TBP is challenging because it is typically paucibacillary. Acid-fast staining of pericardial fluid has a low sensitivity, ranging from 0% to 42% [8]. Cepheid GeneXpert, a quantitative PCR test for the *M. tuberculosis* complex, offers faster detection but shows considerable variability, being more sensitive on tissue (up to 80%) than on fluid (around 15%) [8]. Culture can take up to three weeks, with variable sensitivity (approximately 53-75%, depending on the medium and handling) [8]. Biochemical testing, including pericardial fluid adenosine deaminase (ADA) and interferon-γ release assays (IGRAs), has reported positive predictive values of 83% and 100%, respectively [9]. Guidelines recommend a combined diagnostic approach to improve overall yield, including consideration of pericardial biopsy [4].

Here, we present a case of TBP with cardiac tamponade in a patient receiving infliximab, which led to a fulminant inflammatory response and local spread of disease. This case highlights the limitations of pericardial fluid analysis and underscores the critical role of early, invasive tissue sampling in achieving a timely diagnosis.

## Case presentation

A 61-year-old woman presented with progressive shortness of breath, a dry cough, back pain, and shivering. She denied hemoptysis, weight loss, anorexia, or night sweats.

Her past medical history included Crohn’s disease, infliximab-induced generalized exfoliative dermatitis, and chronic kidney disease stage 3. She had recently completed four bimonthly infliximab infusions for Crohn’s disease, with the most recent dose administered two months prior to admission. Additionally, she had undergone several steroid tapering courses for her skin condition, the most recent ending two days before admission.

The patient was born in India and moved to the UK at the age of five. She is a lifelong non-smoker with no known exposure to TB and no family history of TB. She had received the BCG vaccine as a child. A prior IGRA test, performed in September 2021 before starting immunosuppressive therapy for Crohn’s disease, was negative.

On presentation, she appeared unwell, clammy, and diaphoretic. She was tachypneic, with reduced air entry at both lung bases, muffled heart sounds, raised jugular venous pressure, and pitting edema over both mid-shins. Vital signs on admission are summarized in Table 1.

**Table 1:** Vital signs on admission

Parameter	Patient value	Reference range
Heart rate (beats per minute, bpm)	114	60-100
Respiratory rate (breaths per minute)	32	12-20
Oxygen saturation on room air (%)	92	≥95
Blood pressure (mmHg)	98/74	91-129/61-89
Temperature (°C)	37.6	36.1-37.2

**Figure 1:**
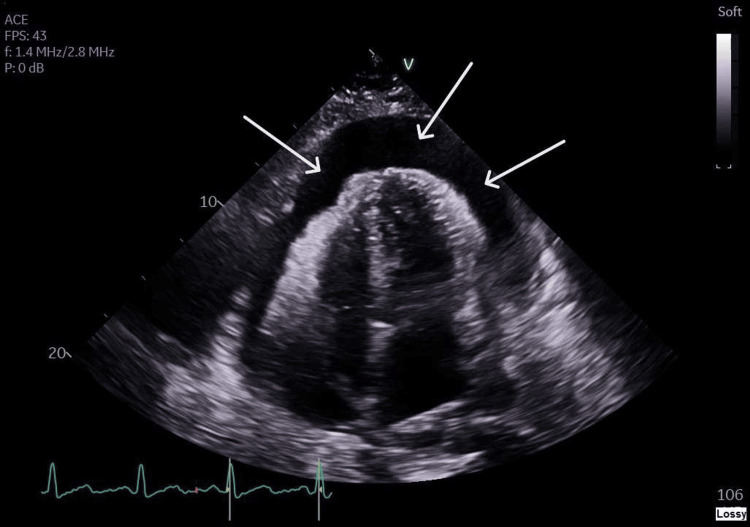
Transthoracic echocardiography, apical four-chamber view, demonstrating a global pericardial effusion with RV collapse White arrows indicate the pericardial effusion. RV, right ventricular

**Figure 2:**
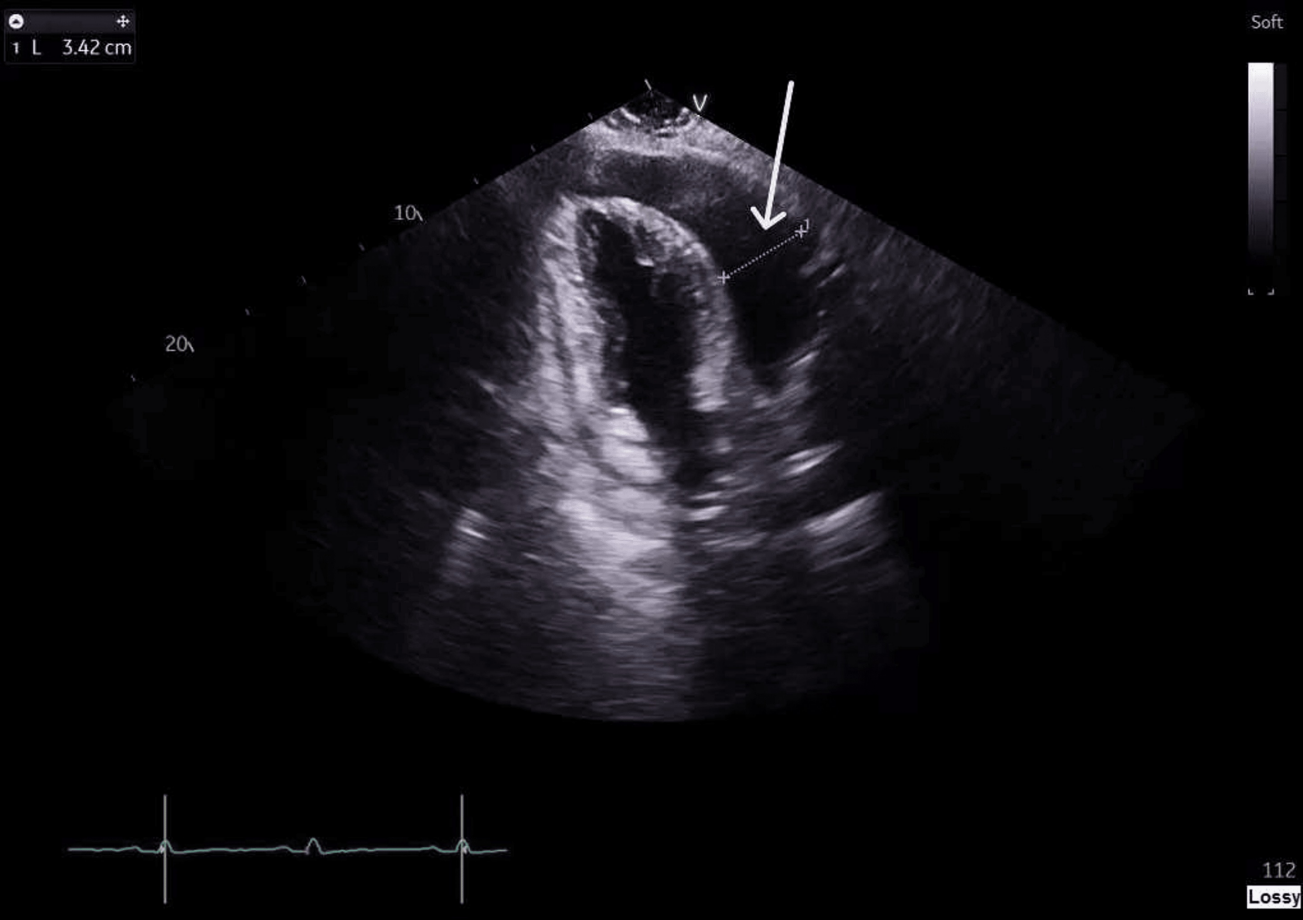
Transthoracic echocardiography, apical two-chamber view, showing the anterior dimension of the pericardial effusion measuring 3.42 cm The white arrow indicates the effusion at its greatest dimension.

**Figure 3:**
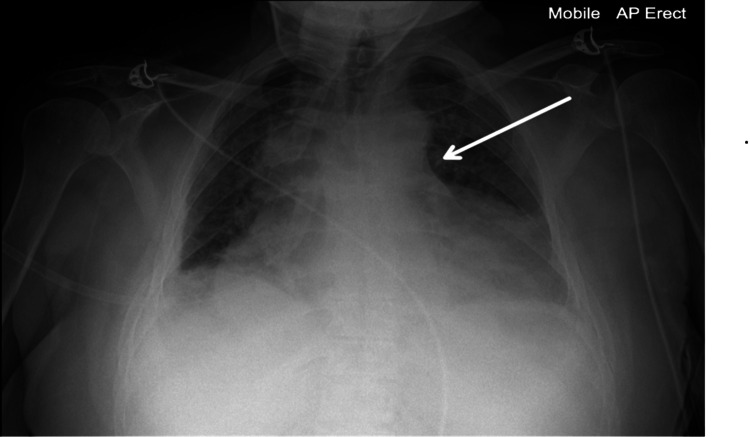
Chest X-ray showing a bulky mediastinum with poorly defined heart borders and bilateral pleural effusions The white arrow points to the widened mediastinal borders.

Bedside focused echocardiography identified a large pericardial effusion with evidence of right ventricular (RV) and right atrial (RA) collapse. A formal echocardiogram confirmed cardiac tamponade, with an RV diastolic dimension of 3.42 cm and an RA dimension of 1.7 cm (Figure 1, Figure 2). Emergency pericardiocentesis was performed, draining 1.2 L of hemorrhagic fluid. A chest X-ray showed a bulky mediastinum with poorly defined heart borders (Figure 3).

Despite drainage, the patient remained febrile and intermittently hypotensive, requiring vasopressor support. She was admitted to the ICU for organ support. Laboratory investigations revealed a marked inflammatory response, hyponatremia, and hypoalbuminemia. Results are summarized in Table 2.

**Table 2:** Laboratory results after drainage of pericardial effusion ALP, alkaline phosphatase; ALT, alanine aminotransferase

Test	Patient value	Reference range
White cell count (×10⁹/L)	>20	4.0-11.0
C-reactive protein (mg/L)	>300	<5
Sodium (mmol/L)	126	135-145
Creatinine (µmol/L)	130	60-110
Urea (mmol/L)	6	2.5-7.8
Bilirubin (µmol/L)	20	<21
ALT (U/L)	17	<41
ALP (U/L)	65	30-130
Albumin (g/L)	26	35-50

**Figure 4:**
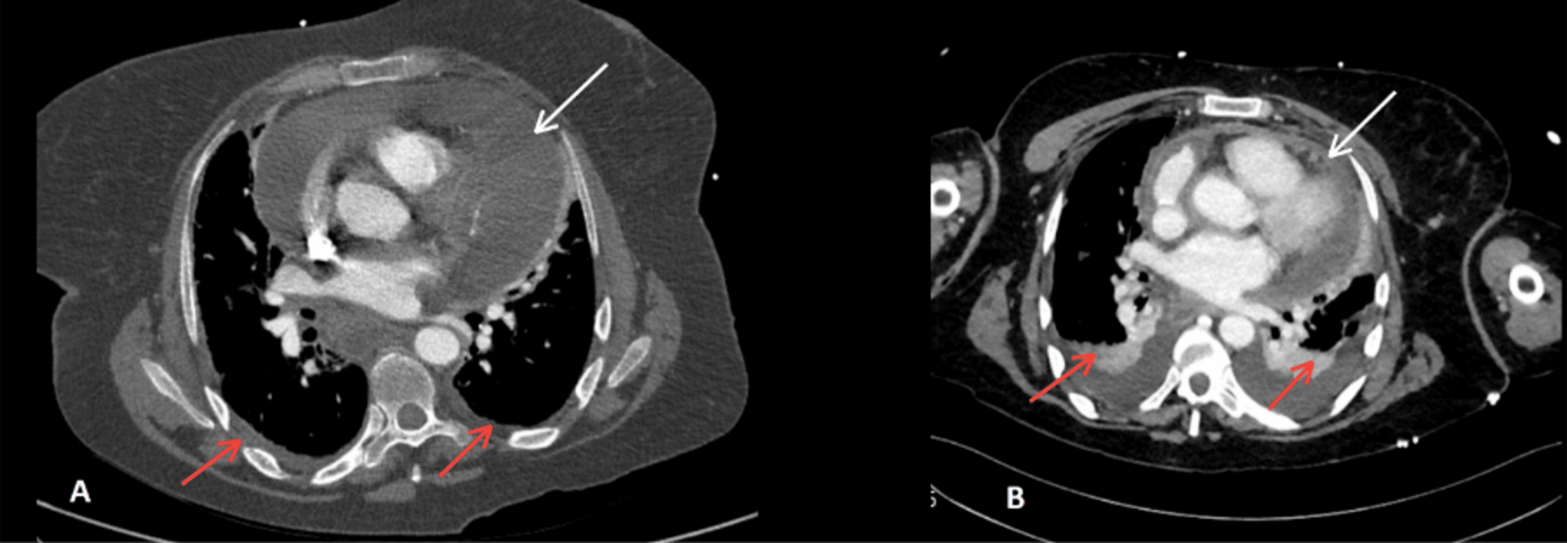
Chest CT angiograms - vascular windows (A) CT aortogram on Day 1 showing a large pericardial effusion (white arrow) and minimal bilateral pleural effusions (red arrows). (B) CT pulmonary angiogram on Day 2 showing interval reduction of the pericardial effusion (white arrow) with mild pleural effusions (red arrows).

A CT aortogram, requested for suspected aortic dissection, revealed a large pericardial effusion, minimal bilateral pleural effusions, and diffusely scattered bilateral centrilobular pulmonary nodules suggestive of inflammatory or infectious etiology, along with prominent intra- and extra-thoracic lymph nodes (Figure 4).

**Figure 5:**
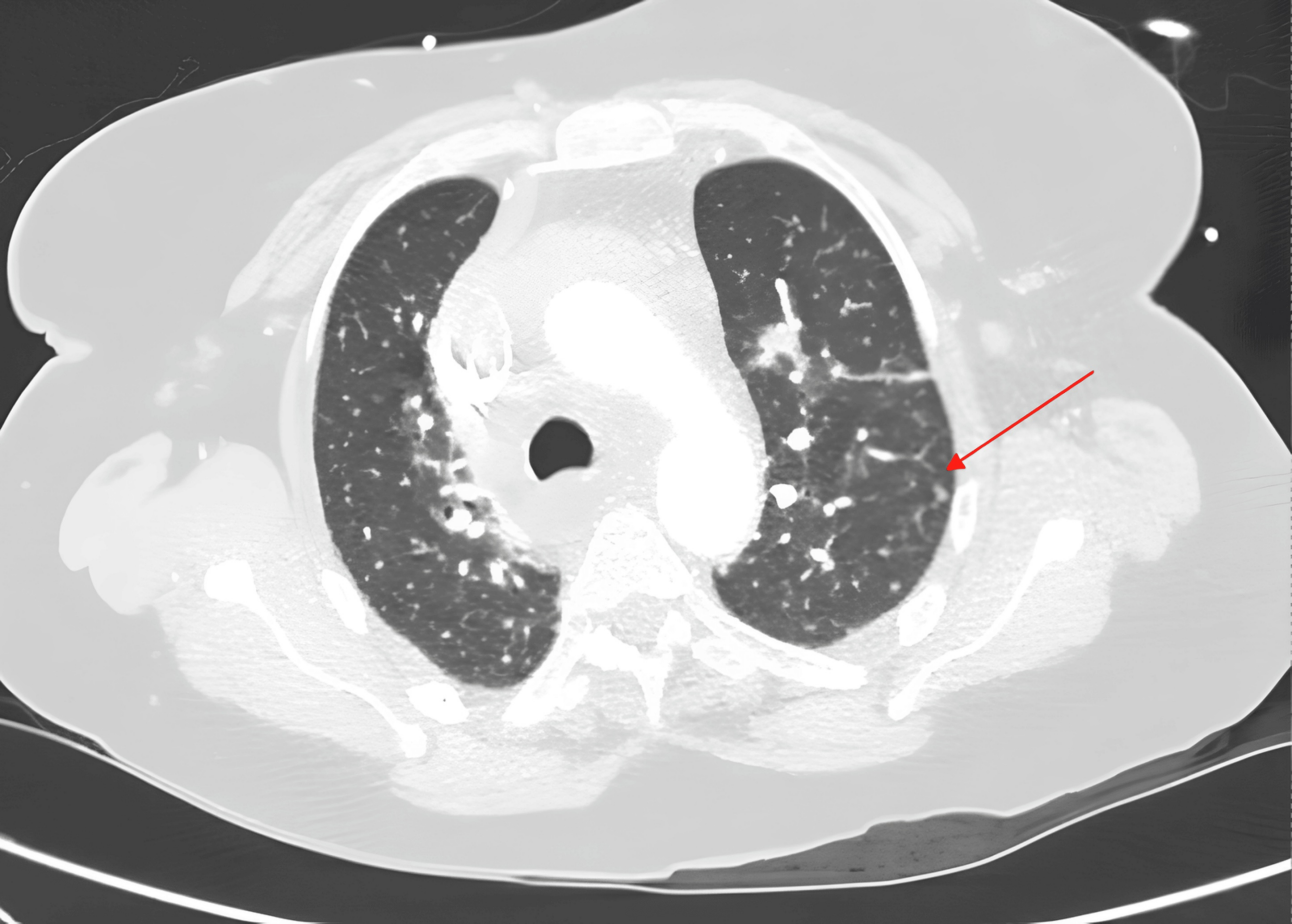
CT aortogram - lung window showing diffusely scattered bilateral centrilobular pulmonary nodules (red arrow)

Multiple prominent lymph nodes were observed in the supraclavicular, bilateral axillary, and mediastinal regions, the largest measuring 10 mm in the left axilla. A subsequent CT scan of the abdomen and pelvis excluded malignancy and demonstrated interval reduction of the pericardial effusion (Figure 5).

Initial pericardial fluid analysis demonstrated mixed inflammatory cells by cytology, with no organisms seen on Gram stain. Once TB was suspected, the pericardial fluid sample was cultured. Acid-alcohol fast bacilli (AAFB) smear was negative, but MTB was isolated on fluid culture, although these results became available several weeks after diagnosis was confirmed by Cepheid GeneXpert PCR.

In this case, ADA and IGRA testing were not performed. IGRA was not pursued because it cannot distinguish latent from active TB in high-risk, immunosuppressed individuals, and ADA was not measured during the emergent pericardial procedure, representing a missed diagnostic opportunity.

Biopsy of the left axillary lymph node was abandoned due to reduced size and difficult location. The patient was transferred from the ICU to the cardiology ward for further evaluation of the pericardial effusion. She remained breathless, requiring 1 L of supplemental oxygen to maintain oxygen saturation. Her blood pressure was 175/110 mmHg, heart rate 110 bpm, and temperature 38.1 °C.

During admission, she received 10 days of intravenous piperacillin-tazobactam for suspected severe pneumonia. Although her inflammatory markers were trending downward, she continued to experience intermittent fevers and showed no clinical improvement. Urine and blood cultures were negative, and she tested negative for both COVID-19 and influenza antigens. Given clinical suspicion for TB, she was referred to the respiratory team.

The respiratory team recommended sampling other lymph node groups and repeating TB cultures along with GeneXpert testing. Bronchoscopy with endobronchial ultrasound-guided biopsy (EBUS) was advised.

**Figure 6:**
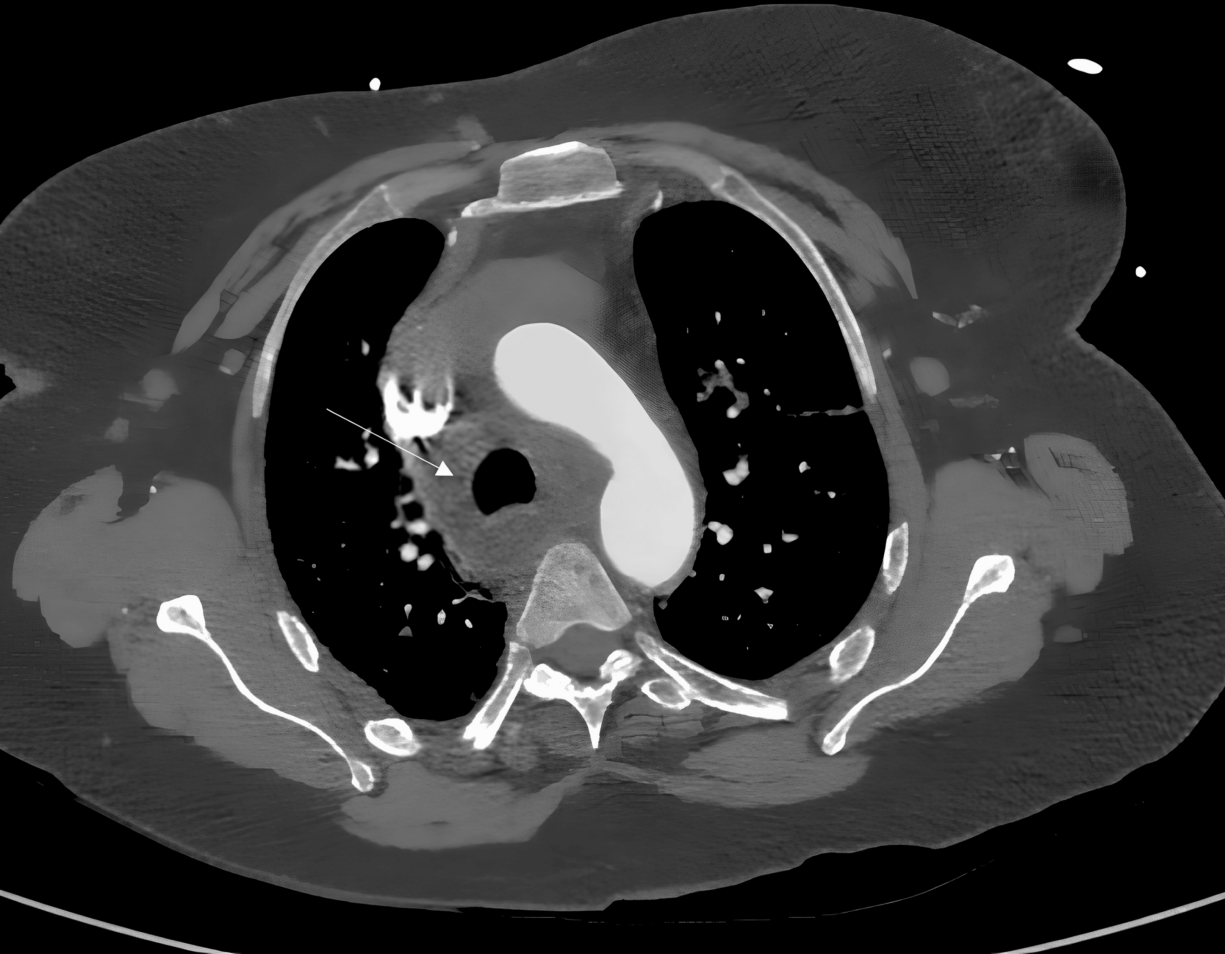
CT pulmonary angiogram - mediastinal window showing an enlarged right paratracheal lymph node (white arrow)

Cepheid GeneXpert testing of a right paratracheal lymph node (Figure 6) detected the *M. tuberculosis* complex with no gene mutations indicating rifampicin resistance. The TB culture of the sample was AAFB smear positive. The National Mycobacterium Reference Service reported Lineage 1 *M. tuberculosis* susceptible to all first-line agents: rifampicin, isoniazid, pyrazinamide, and ethambutol. EBUS bronchial wash samples were negative for MTB by Cepheid GeneXpert, AAFB smear, and TB culture. National guidance recommends that all patients diagnosed with MTB infection be tested for HIV; this patient was HIV negative.

After two weeks of admission, the patient was commenced on quadruple anti-tuberculous therapy (HRZE regimen: isoniazid, rifampicin, pyrazinamide, and ethambutol). Infliximab and other immunosuppressants were withheld. She was discharged clinically stable with outpatient follow-up in the TB clinic and infection control precautions in place.

## Discussion

TB remains a major global health issue, with an increasing overall incidence, reversing the long-term decline observed until 2020. The majority of cases occur in Southeast Asia and Africa, while incidence in Europe remains comparatively low [10]. However, UK epidemiological studies have shown a rising incidence, particularly among patients born outside the UK. Immunosuppression contributes significantly to TB risk in this cohort [10]. Although rare, pericardial involvement is an important sequela of TB, associated with substantial morbidity and mortality [11].

Our case demonstrates several notable divergences from typical TBP presentations and provides valuable insights into the literature. First, the patient presented with acute cardiac tamponade rather than the classically described insidious onset of pericardial effusion. This uncommon presentation challenges the typical natural history described in previous studies [12].

Second, TBP occurring in the context of anti-TNF therapy in an HIV-negative patient is less frequently reported than HIV-associated TBP. This case contributes to the understanding of immunosuppression-related TBP beyond the predominant focus on HIV as a risk factor [2,8].

Third, the transient left ventricular systolic dysfunction observed after pericardial drainage is an uncommon but clinically important finding. Most literature on TBP emphasizes effusive or constrictive evolution, with acute left ventricular systolic dysfunction post-drainage being infrequently described compared to chronic cardiac compression in restrictive pericarditis [2,13].

The diagnostic challenge arises from nonspecific manifestations and vague cardiopulmonary symptoms. The broad differential diagnosis necessitates a high index of suspicion and a timely, multidisciplinary approach, particularly given the potential for rapid disease progression. Invasive testing is often required when clinical suspicion is high.

## Conclusions

Acute cardiac tamponade secondary to pericardial effusion is extremely rare in TB pericarditis, especially in low-burden countries. This rarity, combined with nonspecific presentation, low-yield initial investigations, and disproportionate mortality, underscores the need for clinical awareness and a high index of suspicion. Clinicians should anticipate the possibility of acute pericardial tamponade and acute heart failure in this context. Urgent bedside echocardiography, pericardiocentesis, and tissue analysis were critical in the management of this patient, particularly in the setting of a severe inflammatory process and obstructive shock.
